# Through the Looking Glass: Measuring Age-Friendliness in the UK

**DOI:** 10.1093/geroni/igaf122.1197

**Published:** 2025-12-31

**Authors:** Hannah Marston, Jeroen Dikken, Verina Waights, Lorna Rouse, Louise Wallace Wallace, Joost van Hoof

**Affiliations:** The Open University, Milton Keynes, England, United Kingdom; The Hague University of Applied Sciences, The Hague, Zuid-Holland, Netherlands; The School of Health, Wellbeing and Social Care, Faculty of Wellbeing, Education & Languages, The Open University, Miltin Keynes, England, United Kingdom; The School of Health, Wellbeing and Social Care, Faculty of Wellbeing, Education & Languages, The Open University, Miltin Keynes, England, United Kingdom; The School of Health, Wellbeing and Social Care, Faculty of Wellbeing, Education & Languages, The Open University, Miltin Keynes, England, United Kingdom; The Hague University of Applied Sciences, Wrocław University of Environmental and Life Sciences, The Hague, Zuid-Holland, Netherlands

## Abstract

Age-friendly cities is an important initiative set out by the World Health Organization and many researchers and policy makers aim to create age friendly environments to support their ageing populations. This presentation will present findings from the UK through the validation of the age-friendly cities and communities questionnaire (AFCCQ-UK) deployed across the UK. The sample (n = 426) completed a survey answering a 23-item measure deployed online in a bid to reach broader audiences throughout the years 2023 and 2024. Employing a life course perspective, the survey targeted people aged 18-60+ years through mailing lists, social media posts, and word of mouth. The AFCCQ-UK demonstrates validity and reliability across two age groups (18-59 and 60+ years) showing for the first time globally that age-friendliness can be measured intergenerationally rather than just among those aged 60+. Respondents demonstrated a moderately positive overall perception of age-friendliness. Data shows no significant differences between the age groups suggesting consistency of age-friendliness across the demographics. However, financial differences were observed to be of influence especially relating to the domains of social participation, and community support and health services. A current case study will illustrate how the AFCCQ-UK is used by stakeholders to measure the age-friendliness across the city of Milton Keynes, a new city developed in the 1960s, to demonstrate how the findings have fed into the strategic priorities of the local authority to make significant changes to improve the city for its residents.

